# Bilateral variant origin of subclavian artery branches

**DOI:** 10.1259/bjrcr.20150429

**Published:** 2016-07-28

**Authors:** Benny Jose Panakkal, Gopalan Nair Rajesh, Harish Babu Parakkal, Gomathy Subramaniam, Haridasan Vellani, Chakanalil Govindan Sajeev

**Affiliations:** ^1^Department of Cardiology, Government Medical College, Kozhikode, India; ^2^Department of Diagnostic and Interventional Radiology, Baby Memorial Hospital, Kozhikode, India; ^3^Department of Radiodiagnosis, Government Medical College, Kozhikode, India

## Abstract

Subclavian artery branching patterns have been studied in cadaveric series and frequencies of the many variations have been documented. However, such variations have been seldom noticed antemortem. Here, we present the case of a very rare type of bilaterally different branching pattern of the subclavian artery.

## Clinical presentation

**Figure 1.**
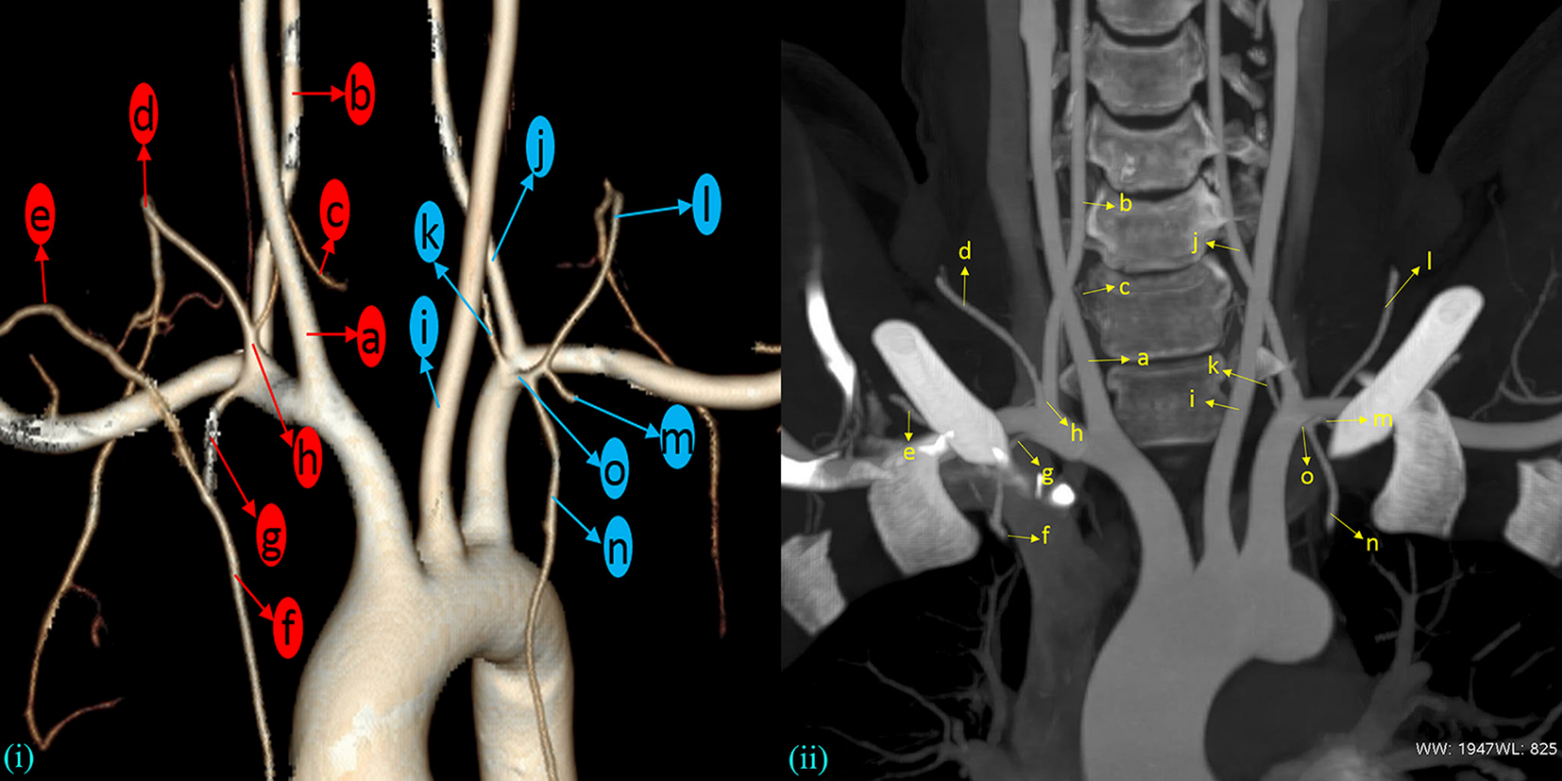
Three-dimensional reconstructed 64-slice CT angiogram (i) and coronal maximum intensity pixel reconstruction (ii) of the aortic arch and its branches. (a) Right carotid artery; (b) right vertebral artery; (c) right inferior thyroid artery; (d) right transverse cervical artery; (e) right suprascapular artery; (f) right internal thoracic artery; (g) common stump branching into (e) and (f); (h) thyrocervical trunk; (i) left carotid artery; (j) left vertebral artery; (k) left inferior thyroid artery; (l) left transverse cervical artery; (m) left suprascapular artery; (n) left internal thoracic artery; (o) common trunk branching into (l), (m) and (n).

A 52-year-old male patient presented to our cardiology department for evaluation of chest pain. He eventually underwent a coronary angiogram during which he was deemed a suitable candidate for coronary artery bypass grafting (CABG). Contrast injection to the left subclavian artery (SCA) to assess the patency of the left internal thoracic artery (ITA) failed to clearly show the artery. Hence, a 64-slice CT angiogram with three-dimensional reconstruction and coronal and axial maximum intensity pixel (MIP) reconstruction was performed, which showed a variant origin of the branches of the left and right SCA (Figure 1 and Supplementary figure). The variation was not the same on both sides. On the left side, the ITA arose from a common trunk that also gave rise to the transverse cervical artery (TCA) and the suprascapular artery (SUS), whereas the inferior thyroid artery (THY) arose directly from the SCA just before the vertebral artery, making it the first branch of the SCA. On the right side, the ITA arose from a common trunk that also gave rise to the SUS. Correspondingly, the right thyrocervical trunk (TCT) had only two branches: the THY and the TCA. Subsequently, the patient underwent CABG without any complications.

## Discussion

**Figure 2.**
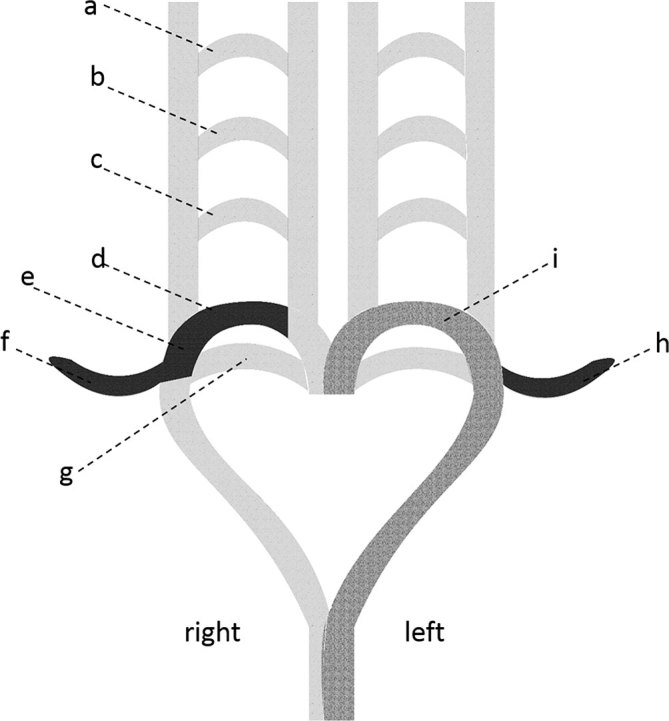
Aortic arches and the development of SCAs. (a) First aortic arch; (b) second aortic arch; (c) third aortic arch (d) fourth aortic arch, which gives rise to the proximal part of the right SCA; (e) part of the dorsal aorta, which gives rise to the middle portion of the right SCA; (f) seventh intersegmental artery, which gives rise to the distal portion of the right SCA; (g) sixth aortic arch; (h) seventh intersegmental artery, which gives rise to the left SCA; (i) arch of the aorta. SCA, subclavian artery.

The left and right SCAs have slightly different embryological origins. The left SCA develops from the left seventh intersegmental artery. The right SCA originates from three structures: the proximal portion from the right fourth aortic arch, the middle portion from the right dorsal aorta in the vicinity and the distal portion from the right seventh intersegmental artery (Figure 2). Although the development of the SCA is widely discussed in the literature, there is little knowledge regarding the embryology of its branching pattern. Similarly, it is known that genetic mutations (22q11 deletion syndromes) can influence the development of the aortic arch and its branches *via* the *TBX1* and fibroblast growth factor genes.^1^ However, it is not yet known whether or not such mutations also affect the branching pattern of the SCA.

**Figure 3.**
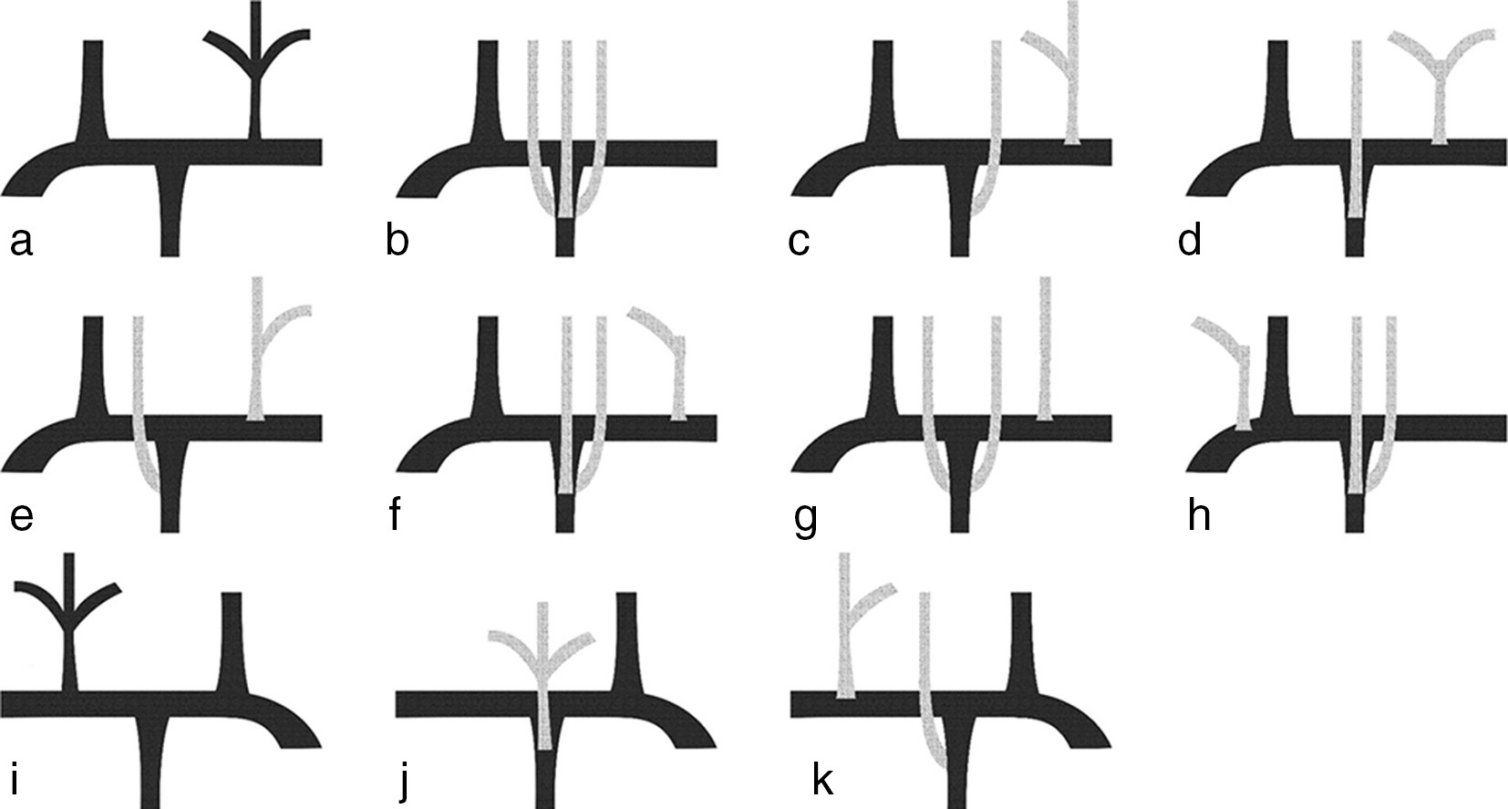
Various branching patterns of the first part of the left (a–h) and right (i–k) SCA. (a) Normal branching pattern of the left SCA—first branch is the VA, second the ITA and third the TCT, which gives rise to the SUS, TCA and THY,; (b) SUS, TCA and THY arising from the ITA; (c) SUS arising from the ITA, and TCA and THY arising from the TCT; (d) TCA arising from the ITA and SUS, and THY arising from the TCT; (e) THY arising from the ITA, and SUS and TCA arising from the TCT; (f) SUS and TCA arising from the ITA, and THY arising directly from the SCA; (g) SUS and THY arising from the ITA, and TCA arising directly from the SCA. (h) Our patient: TCA and SUS arising from the ITA, and THY arising directly from the SCA as its first branch. (i) Normal branching pattern of the right SCA with the pattern analogous to that of normal left SCA; (j) TCT arising from the ITA. (k) Our patient: SUS arising from the ITA, and TCA and THY arising from the TCT. ITA, internal thoracic artery; SCA,subclavian artery; SUS, suprascapular artery; TCA, transverse cervical artery; TCT, thyrocervical trunk; THY, inferior thyroid artery; VA, vertebral artery.

The earliest cadaveric studies of SCA and its branches were performed in 1959,^2^ the data from which suggested that the left ITA along with the four branches of the TCT (TCA, SUS, THY and the ascending cervical artery) was the most common variant combination (7.4%), followed by the left ITA with SUS (2.6%), left ITA with TCA (0.5%), left ITA with THY (0.5%) and left ITA with TCA and SUS (0.1%). In a subsequent study in 1997,^3^ the variant origin of the right ITA was seen only in 5% of cases, whereas that of the left ITA was seen in 30% of cases. Of the variations in the left ITA, the least common was its origin with three branches of the TCT (1%). The most common variation was the left ITA with SUS (16%), followed by left ITA with SUS and TCA (5%), left ITA with THY and the ascending cervical artery (4%) and left ITA with SUS and THY (2%). TCT from ITA is the only reported variation of right SCA branching^4^ (Figure 3). A variant origin of the left ITA antemortem was first reported in 2000 in an angiography-based Croatian study.^5^ In that study, only the presence or absence of variations in branching was documented and the individual types of variations were not taken into account.

This highlights the rarity of the present case report that documents antemortem a combination of both right and left SCA branching variations in the same patient. Theoretically, such variations in branching may lead to future complications such as coronary or shoulder-girdle muscle steal phenomenon and kinking of the artery during CABG.^6^ However, to the best of our knowledge, no such complication has been reported in the literature. It is important to recognize variant anatomy when patients are under consideration for therapeutic interventions such as CABG to help develop safe surgical procedures.

## Learning points

1. A 64-slice CT angiogram with three-dimensional reconstruction should be performed in cases where a variant branching pattern of the SCA is suspected owing to difficult angiographic localization of the same.
2. Such variations, if found preprocedure, may provide additional information to the surgeon to anticipate complications and plan the procedure accordingly.

## Consent

Informed consent has been obtained from the patient for publication of this case report and all images submitted.
